# Atomic Force Microscopy Reveals the Dynamic Morphology of Fenestrations in Live Liver Sinusoidal Endothelial Cells

**DOI:** 10.1038/s41598-017-08555-0

**Published:** 2017-08-11

**Authors:** B. Zapotoczny, K. Szafranska, K. Owczarczyk, E. Kus, S. Chlopicki, M. Szymonski

**Affiliations:** 1 Centre for Nanometer-Scale Science and Advanced Materials, NANOSAM, Faculty of Physics, Astronomy, and Applied Computer Science, Jagiellonian University, Krakow, Poland; 2 Jagiellonian Centre for Experimental Therapeutics, JCET, Jagiellonian University, Krakow, Poland; 3 Chair of Pharmacology, Jagiellonian University, Medical College, Krakow, Poland

## Abstract

Here, we report an atomic force microscopy (AFM)-based imaging method for resolving the fine nanostructures (e.g., fenestrations) in the membranes of live primary murine liver sinusoidal endothelial cells (LSECs). From data on topographical and nanomechanical properties of the selected cell areas collected within 1 min, we traced the dynamic rearrangement of the cell actin cytoskeleton connected with the formation or closing of cell fenestrations, both in non-stimulated LSECs as well as in response to cytochalasin B and antimycin A. In conclusion, AFM-based imaging permitted the near real-time measurements of dynamic changes in fenestrations in live LSECs.

## Introduction

First identified in liver sinusoids more than 60 years ago, fenestrations are transmembrane pores with sizes ranging from 50 to 200 nm^1–3^ that accumulate in groups of up to several tens in sieve plates^4–6^. Liver sinusoidal endothelial cells (LSECs) exhibit unique features characterized by the presence of fenestrations and lack of basement membrane; this morphology is strongly associated with the sieve function of these cells^2^. Interestingly, the diameter and number of fenestrations in LSECs change in response to treatment with various agents, e.g., hormones, drugs, and toxins^1, 7^. Changes in the morphology of fenestrations have been investigated “ex posteriori”; for example, electron microscopy of isolated and subsequently fixed and dehydrated cells has been performed^8–11^. However, due to the invasive nature of sample preparation^12, 13^, contradictory results have often been obtained^1^. Despite a rich knowledge of the structure and function of fenestrations, the dynamic morphology of the fenestrations in live cells remains unknown^14^. Recently, significant progress in imaging techniques has led to the development of super-resolution fluorescence microscopy^15^, scanning near-field optical microscopy^16^, and atomic force microscopy (AFM)^17–19^, which have the potential to facilitate visualization of fenestrations in live LSECs. However, to the best of our knowledge, no clear images of fenestrations in live cells have been reported to date^15, 17, 20^.

Here, we present a novel application of the AFM force imaging mode based on fast acquisition of the force versus distance (FD) curves in every pixel of the scanned area. Reconstructing the topographic image from thousands of FD curves collected in milliseconds permits not only the high-resolution imaging of detailed topographical structures on LSECs but also the observation of rapid alterations in fenestrations morphologies in live LSECs. Because of the flat, thin appearance of LSECs, short FD curves, i.e., below 200 nm could be used. Therefore, when a relatively small area was scanned, high-resolution images could be acquired rapidly (time per frame up to 45 s for a single sieve plate).

## Results and Discussion

**Figure 1:**
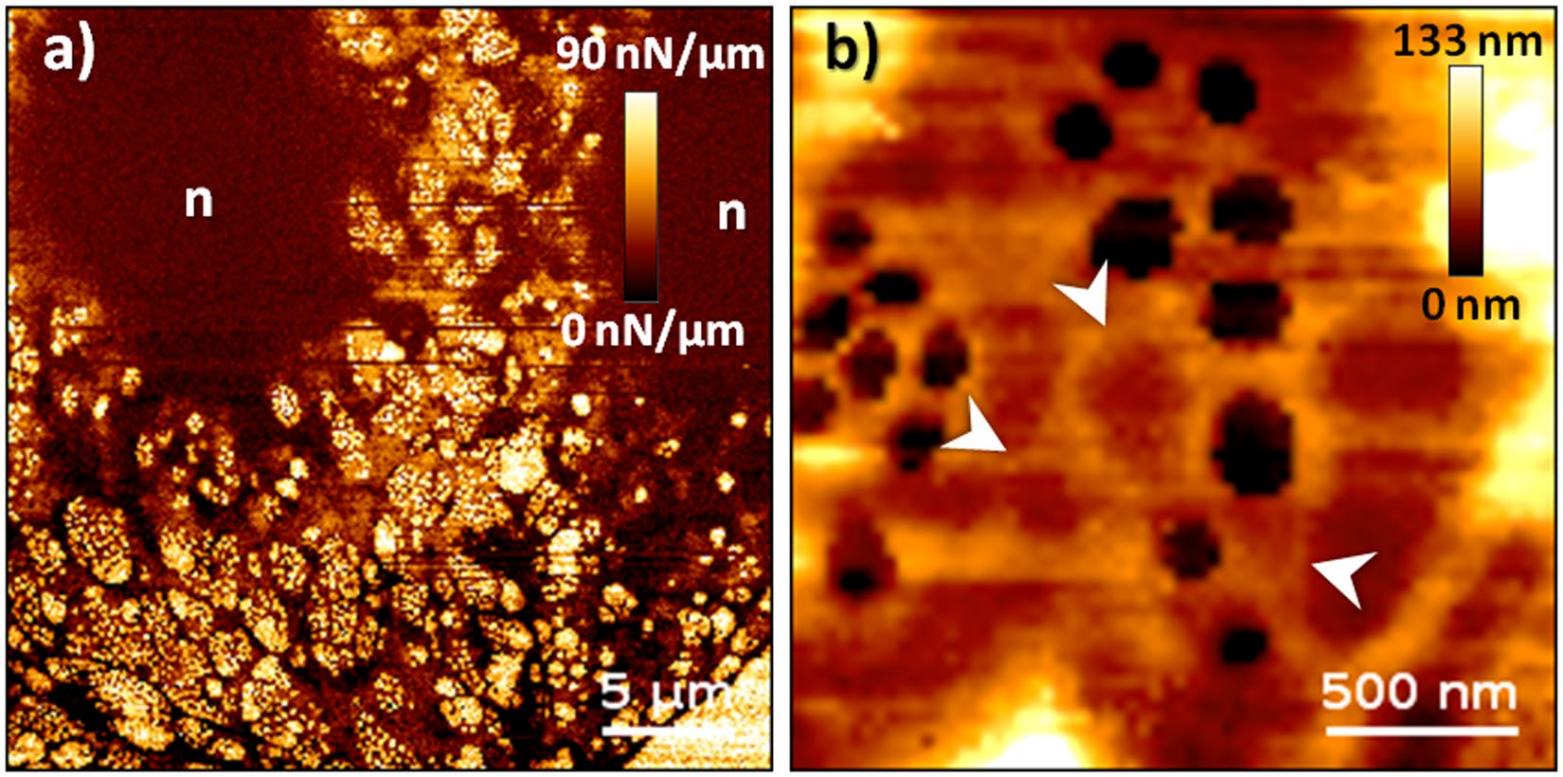
Live LSECs visualized using fast FD-based AFM imaging mode. (**a**) Stiffness alterations revealing the fenestrated morphology of cells. Bright and dark colours represent stiff and soft regions, respectively. Several sieve plates filled with fenestrations are observed in the peripheral regions of cells, i.e., out of the nucleus zone denoted by “n”. (**b**) High-magnification topographical images of single sieve plates allowing estimation of the fenestration diameters in live cells (Supplementary Fig. 1). Fenestrae-associated cytoskeleton rings were observed as brighter structures, some of which were filled with membranes (white arrowheads). The cell height in the sieve plate zone was in the range of 40–50 nm. Image size and resolution: (**a**) 30 × 30 µm, 256 × 256; (**b**) 3 × 3 µm, 64 × 64. Maximum loading force: (**a**) 1 nN; (**b**) 600 pN, image reconstructed for 400 pN.

Imaging based on force spectroscopy enabled us to construct images containing detailed information regarding both the topography and nanomechanical properties of whole live LSECs as well as s single sieve plate (Fig. 1).

The low-magnification stiffness image of live LSECs is shown in Fig. 1a, in which the extremely porous cell structure is clearly observed. Such images are possible because of the large contrast difference between the inner regions of the fenestrations (the AFM tip probes a stiff glass slide; bright points) and the soft cell body (dark brown). The stiffness values for each pixel of the image were calculated as the slope of the linearized FD curve in a carefully selected scanner position range. The best contrast was obtained if this range extended from 0 to 5–10 nm, i.e., in the narrow range close to the maximum deflection of the cantilever used in the experiment (for more details see Methods). Topographic images could be extracted from the same set of force distance curves for any loading force values. For example, a high contrast reconstruction obtained for the loading force of 400 pN is shown in Fig. 1b. Due to the dynamic rearrangement of the fenestrations (see the next paragraph) and the tip apex size of 20–60 nm, it is quite challenging to accurately measure the fenestration diameters in live LSECs. Nevertheless, a good distribution of the fenestration diameter can be made based on high spatial resolution images of fenestrations from several sieve plates. The histogram of 985 experimentally measured diameters allowed us for determination of the statistically most probable value which was equal to 180 nm ± 41 nm (see Supplementary Fig. 1). Moreover, we were able to identify some of the stress fibres located next to the fenestrations (Fig. 1b), in agreement with previous reports^18, 21–24^. We found that the ability to visualize the cytoskeleton structures (fenestrae-associated cytoskeleton rings [FACRs])^1, 25^, or stress fibres was strongly related to the number of analysed FD curves per image area, the applied loading force, and the speed of FD curve acquisition (Supplementary Fig. 2).

**Figure 2:**
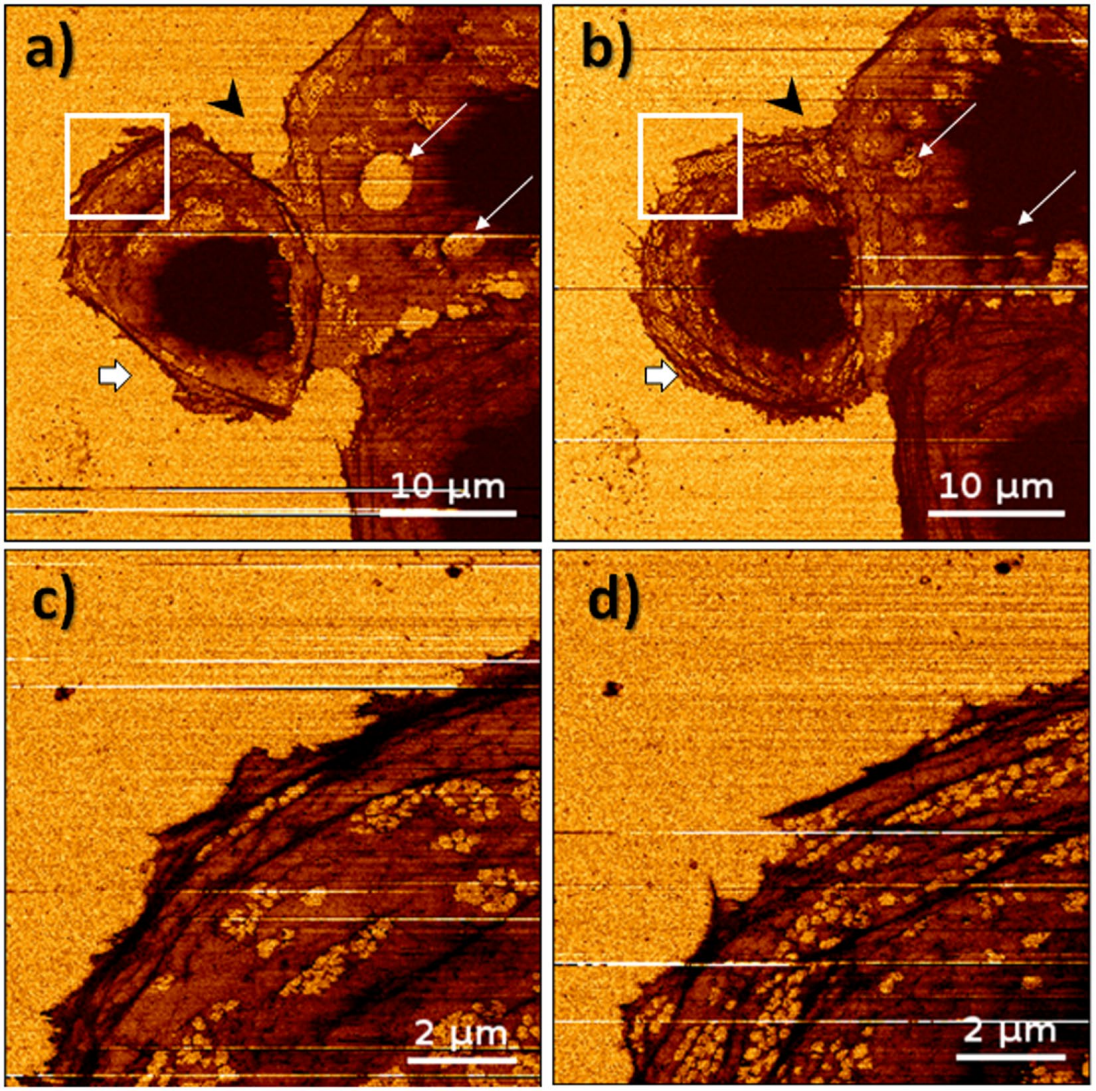
LSECs visualized using FD-based AFM imaging mode, where stiffness parameter is presented. (**a**,**b**) Spreading of the cell on a glass slide occurs (thick arrows) after approximately 65 minutes of measurements leading to better connection between cells (black arrowhead). Regeneration of micrometre size holes is observed (thin arrows). (**c,d**) High-magnification images were made meantime revealing detailed changes in the structure of the cytoskeleton leading to the formation of new sieve plates and fenestrations. Images size and resolution: (**a,b**) 50 × 50 µm, 256 × 256, (**c**,**d**) 10 × 10 µm, 256 × 256. Maximum loading force: 700 pN.

The main results of our work demonstrate that live LSEC morphology could be studied under AFM for several hours practically in real time starting from the time of cell seeding on the glass slide (see Supplementary Animations 1–4). In Fig. 2 we show morphology of three live LSECs after 6 hours from the seeding on a glass coverslip (Fig. 2a) and approximately 65 minutes later (Fig. 2b). The overall appearance of cells changes, i.e., the cell surface area is larger leading to better interconnection between the cells, which is characteristic for the endothelial cells (Fig. 2, black arrowhead). We notice the disappearance of the micrometre size holes in the cell membrane (Fig. 2, white arrowheads) and the increasing number of fenestrations. Rarely, we identify LSECs (Fig. 2a,b, bottom cell), which present thick stress fibres. We noticed that those cells present less fenestrated appearance. The number density of fenestrations varies with time, especially for peripheral parts of the cell, where the actin cytoskeleton rebuilds rapidly (Fig. 2c,d).

To ensure that such high-speed imaging did not cause any alterations in the cell membrane, we performed time-dependent control experiments in which the selected area was sequentially scanned (see Supplementary Animations 1 and 2, reconstructed from 6 and 14 AFM images, respectively). We noticed that after 16–24 h, the number of fenestrations did not change significantly, particularly in regions close to the interconnections of few cells (Supplementary Animations 1 and 2); therefore, further experiments were performed within that time frame.

Through visualization of fenestrations at high resolution and observation of actin cytoskeleton rearrangement in real time, we could investigate the responses of live LSECs to different agents, including cytochalasin B, an actin-disrupting agent, and antimycin A, an inhibitor of mitochondrial electron transport chain (mETC) complex III. In previous reports^26, 27^ describing changes in the numbers of fenestrations in response to these agents, experiments were performed after fixation and dehydration of the cells.

**Figure 3:**
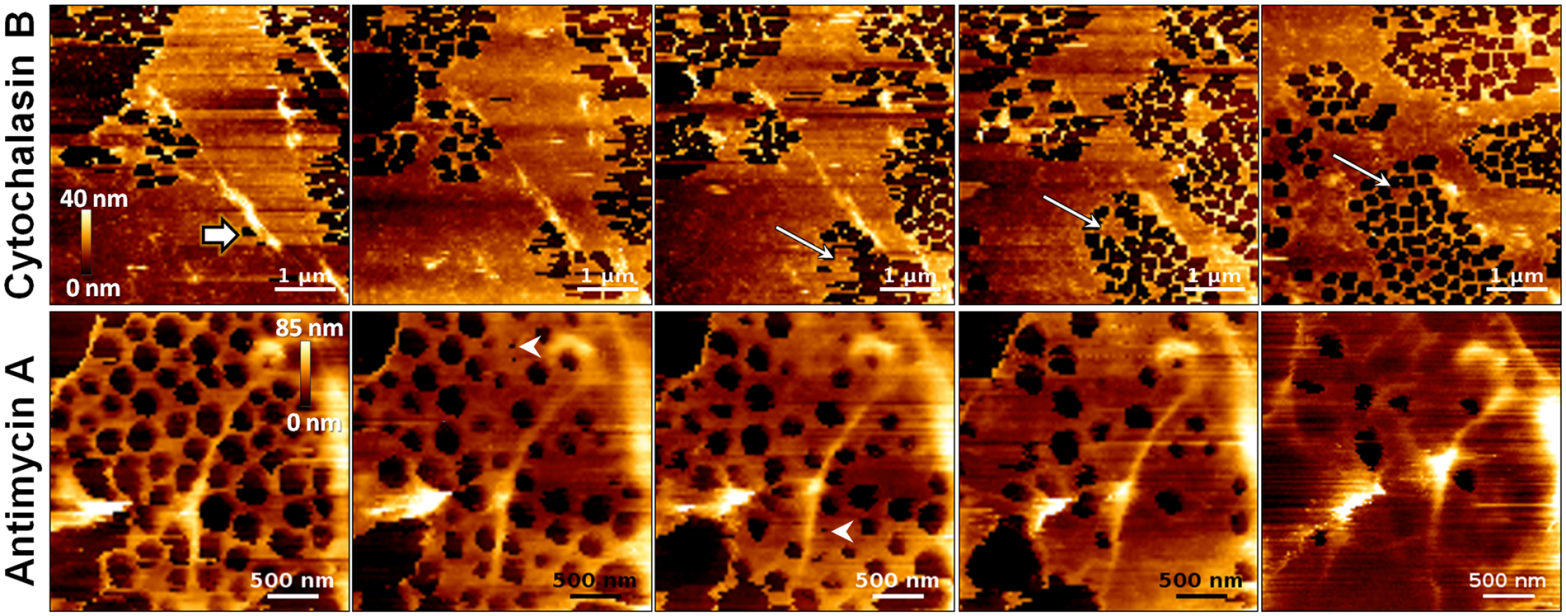
Topographical images of the selected area of live LSEC exposed to cytochalasin B and antimycin A (upper and lower row, respectively). Effects of 21 µmol/dm^3^ cytochalasin B were measured 2, 4, 6, 11, and 25 min after the addition of the compound (upper row). Each single image was acquired within 45 s (Supplementary Animation 3). Rapid increases in fenestration numbers were observed after the treatment with cytochalasin B. A newly formed sieve plate appeared 2 min after addition of cytochalasin B (thick, white arrow). The fenestration-forming centre could be distinguished 6 min (white arrow) after addition of cytochalasin B and moved in the direction of sieve plate expansion. Image size, resolution, and speed of acquisition: 5 × 5 µm, 100 × 100 curves, and 100 µm/s, respectively. Topography of the cell membrane of live LSECs in response to 1 µg/mL antimycin A was measured 0, 8, 15, 22, and 60 min after addition of the compound (lower row). Single images were acquired within 3 min (Supplementary Animation 4). The process for decreasing the fenestration size, leading to loss of fenestrations, was observed. Arrowheads indicate fenestrations with diameters of less than 80 nm just before cell membrane fusion. Image size, resolution, and speed of acquisition: 3 × 3 µm, 90 × 90 curves, and 50 µm/s, respectively.

Figure 3 shows selected images from a 25 min experiment in which we investigated changes in the morphologies of selected LSEC areas after the addition of cytochalasin B. Single images were acquired within a time frame as short as 45 s (Supplementary Animation 3, based on 34 AFM images). We observed rapid widening of the existing sieve plates and an increase in the number of fenestrations (changes in the numbers of fenestrations and the porosity over time are summarized in Supplementary Fig. 4). Furthermore, we observed newly formed

sieve plate after 2 min of incubation with cytochalasin B (Fig. 3, thick, white arrow). After an additional 3 min, we identified the structure corresponding to the previously reported fenestrae-forming centre (FFC) on the sieve plates^25^. We noticed that the sieve plates expanded over time and that the FFC moved simultaneously in the same direction as the number of fenestrations increased (Fig. 3, white arrows). Thus, these observations confirmed the role of FFCs in the formation of new fenestrations^1, 25^. Moreover, the imaging method presented here may allow us to investigate the sequences of cellular events linked to the formation of fenestrations, effectively in real-time.

To further emphasize the potential of the method, we evaluated changes in the numbers and diameters of fenestrations following the treatment with antimycin A, an mETC complex III inhibitor. Previous experiments using scanning electron microscopy showed that the diameters and numbers of fenestrations decreased after 30 min of antimycin A treatment, leading to nearly complete loss of fenestrations after 60 min^26^. Antimycin A affects also FACR structures, leading to shortening of the actin fibres and thus to loss of fenestrations. Based on our ability to visualize time-dependent alterations in morphology, we observed shrinkage and gradual contraction of fenestrations, leading to their closing over time (Fig. 3, lower row and Supplementary Animation 4). Interestingly, FACRs were partially identified in the image, even after the loss of fenestrations. This suggested that FACR and other actin-binding proteins may be involved in maintaining the fenestrations.

## Conclusions

In conclusion, we have shown that the specific scanning conditions permitted by our new AFM-based force spectroscopy and imaging methods (namely, exploring the capabilities of the JPK Quantitative Imaging Mode) facilitated visualization of the dynamic morphology of live LSECs, reaching a spatial resolution well below 100 nm and yielding images within a short time. This experimental approach allowed us to study changes in LSEC fenestration numbers and diameters in response to cytochalasin B and antimycin A. Thus, this new approach permitted fenestrations to be visualized in live LSECs in near real time observation. This novel AFM method will facilitate more in-depth research on LSEC morphology and function, revealing the true dynamic processes in live cells and the time-dependent cellular responses to adverse (toxic) or therapeutic agents.

## Methods

### Cell isolation

Murine LSECs were isolated as previously described^17^. After isolation, LSECs were seeded on uncoated glass coverslips in EGM-2 medium (Lonza) at 37 °C in an atmosphere containing 5% CO_2_. LSECs were cultured for 1–24 h and then examined using AFM.

### AFM

**Figure 4:**
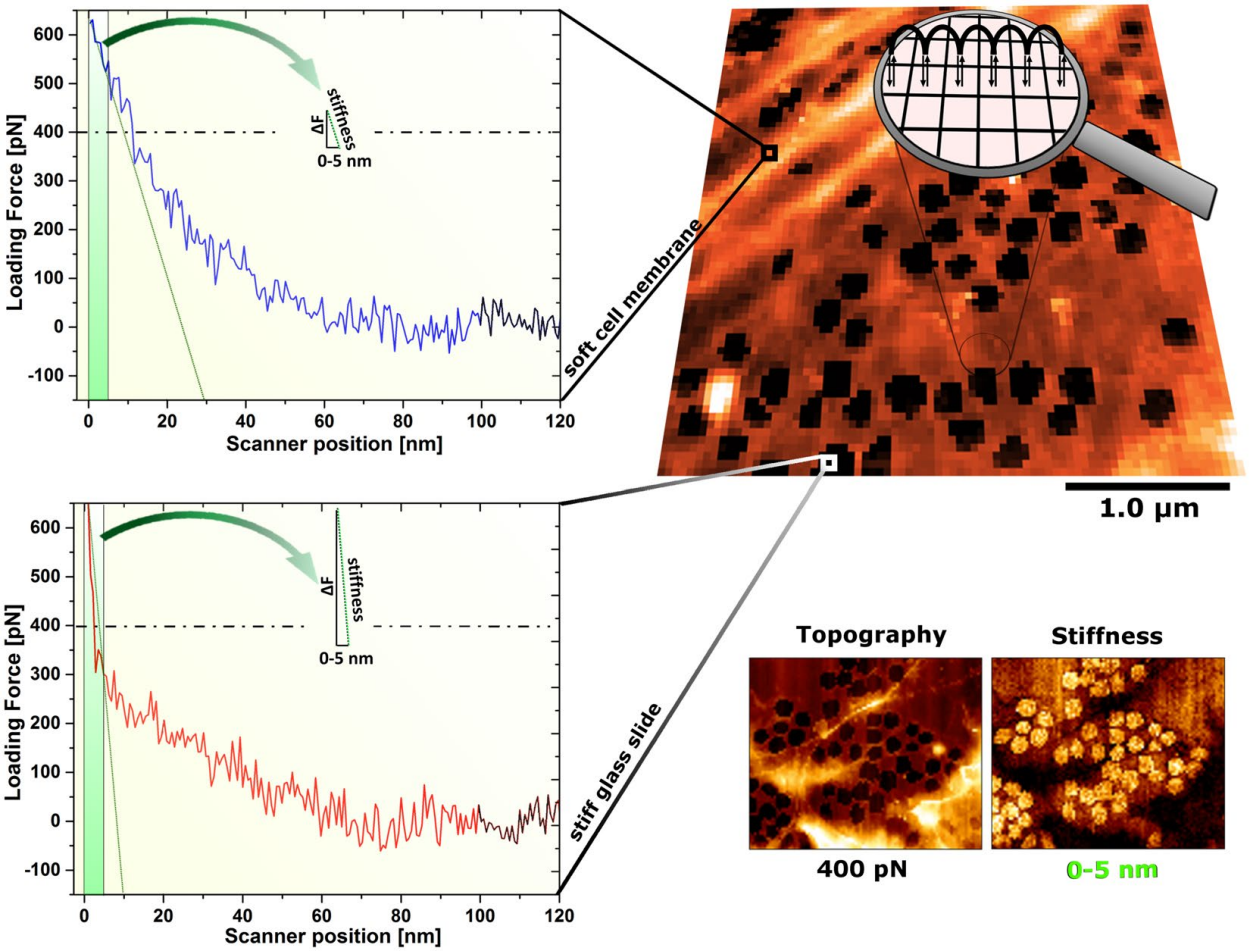
The principle of application of fast FD-based AFM imaging mode used to resolve fenestrations in live LSECs. An image is reconstructed from thousands of FD curves each acquired within milliseconds (top right). Two representative FD curves from soft cell and the inner regions of fenestrations are shown. To ensure high contrast between fenestrations and the cell membrane, an appropriate high loading force of 400 pN was necessary. The dashed lines on the graph indicate the loading force used to reconstruct the presented topographical image. Similar high contrast in stiffness imaging was observed in the narrow range of the FD curves corresponding to the maximum loading force used in the experiment (obtained at the *z* scanner position denoted as 0), namely for the scanner positions from 0 to 5 nm on the extended fragment of the FD curves (green area on the graph). High-speed imaging is possible due to shortening of the *z* scanner movement (horizontal axis on the graph) to the minimum value, where there is no interaction between the tip and the sample. The baseline correction occurred after each FD curve using a curve region with maximum scanner position at 20 nm (mean value of the black-coloured curve region).

Measurements were conducted in EGM-2 medium at 37 °C under ambient conditions using a Nanowizard 3 AFM system (JPK Instruments). Measurement of LSECs was conducted within 3 h. We perform experiments using fast FD-based AFM imaging mode (Quantitative Imaging; JPK Instruments), in which thousands of FD curves were collected and translated into topography and stiffness images. Because of the flat and thin morphology of LSECs, acquired FD curves had lengths of 100–400 nm. To ensure good stability of loading force values and minimize lateral forces during the measurement period, continuous correction of the baseline was necessary. The baseline correction was calculated automatically by the AFM software after obtaining each FD curve using the mean value from the curve region of the maximum z scanner range (20 nm), where there was no interaction between the tip and the sample (black-coloured curve region in Fig. 4). Topographical images were translated from the chosen trigger force selected from the acquired FD curves. Linear fit to the selected area of the FD curve was used to calculate stiffness. In our experiments, we noticed that the highest contrast allowing identification of fenestrations was reached when a narrow area, based on the maximum cantilever deflection, was analysed. Depending on the cell body thickness, tip size, and tip geometry, different values of both maximum loading force (from 500 to 1000 pN) and *z* scanner position (from 0 to 5–10 nm) gave the best imaging conditions. We carried out experiments using a tip with a radius of 20 nm mounted on cantilevers with a spring constant of 0.7 N/m. The following settings were used for cytoskeletal imaging: *z* scanner range (Fig. 4, magnifying glass, black arrows) of 400 nm, tip velocity during acquisition of the extended fragment of the FD curve of 30–50 μm/s, additional *z* scanner retraction (Fig. 4, magnifying glass, black arches) from the recorded area of 400–500 nm. Additionally, the following settings were used for high-magnification images in experiments determining the dynamics of LSECs treated with cytochalasin B: *z* range of 100 nm, tip vertical velocity of 100 μm/s, and additional retraction of 30 nm. The settings for imaging after treatment with antimycin A were as follows: *z* range of 150 nm, tip velocity of 50 μm/s, and additional retraction of 50–100 nm.

The obtained images of the topography and stiffness were analyzed using JPK Data Processing Software. Additionally, ImageJ software was used for calculation of fenestration diameters and porosity^28^.

### Treatment of LSECs with cytochalasin B and antimycin A

During AFM measurements, cytochalasin B (from *Dreschslera dematioi*; Sigma Aldrich, Germany) was added at a final concentration of 21 µmol/dm^3^ in EGM-2 medium, and measurements were continued immediately. Similarly, antimycin A (from *Streptomyces* sp.; Sigma Aldrich) was added to a final concentration of 1.9 µmol/dm^3^. Stock solutions of toxins (1000 × in DMSO) were prepared as described elsewhere^27^.

## Electronic supplementary material

Supplementary Information

Supplementary Animation 1

Supplementary Animation 2

Supplementary Animation 3

Supplementary Animation 4
