# Supplementary material for: Atomic Force Microscopy Reveals the Dynamic Morphology of Fenestrations in Live Liver Sinusoidal Endothelial Cells
Source: Sci Rep. 2017 Aug 11;7:7994. doi: 10.1038/s41598-017-08555-0 (PMC5554186; doi:10.1038/s41598-017-08555-0)
Supplement: Supplementary file 1 — Supplementary Information [file 41598_2017_8555_MOESM1_ESM.pdf]

AFM Reveals Dynamic Morphology of Fenestrations in Live Liver Sinusoidal Endothelial Cells

B. Zapotoczny[1], K. Szafranska[1,2], K. Owczarczyk[1], E. Kus, S.K. Chlopicki[2,3], M. Szymonski[1]

Supplementary Information

1. Evaluation of the mean fenestration diameter,
2. Visualization of the actin fibres and fenestrations in live LSECs,
3. Dynamic changes in the porosity and number of fenestrations after treatment with cytochalasin B and antimycin A,
4. Description of the supplementary animations.

Supplementary Data 1 | *Evaluation of the mean fenestration diameter*

Several high-magnification AFM images of the topography of the peripheral parts of live LSECs were collected and used for characterisation of the cell sieve plates and the fenestrations within. The distribution of the 985 experimentally measured fenestration diameters is shown in the histogram (**Supplementary Fig. 1**). The distribution was fitted with a Gaussian function in order to determine the most probable value of the diameter which was ~180 nm ± 41 nm. The result was 20-30 nm larger than the mean fenestration size, typically reported for 1% glutaraldehyde fixed cells[1]. The step size in analysis of the images was below 30 nm. The AFM tip apex radius was ~20 nm. The step size and low stiffness of the cell membrane combined with continuous rearrangement of the live LSEC actin cytoskeleton limited a correct visualization of the fenestrations smaller than 100 nm.

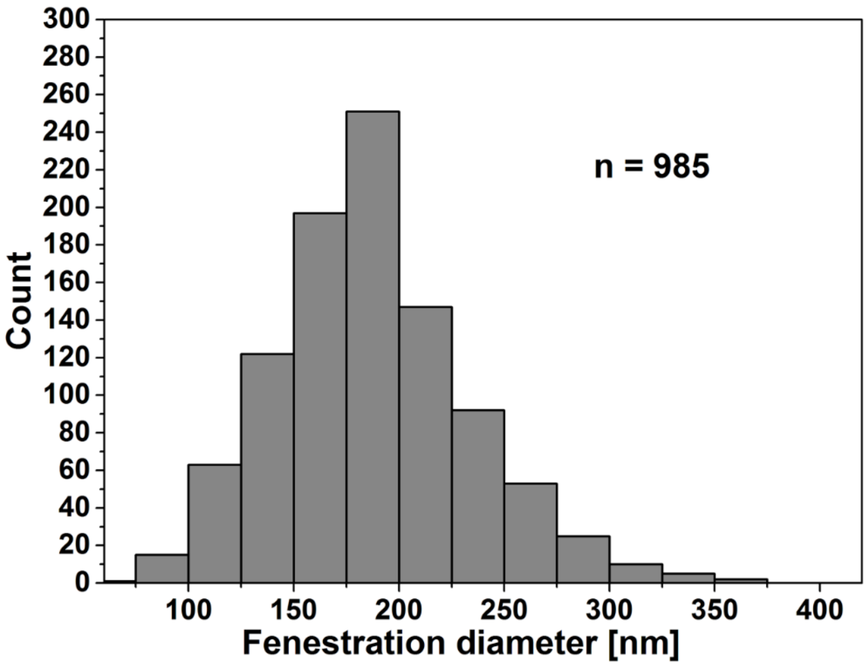

Supplementary Figure 1 | The distribution of the fenestration diameter measured for several high magnification AFM images of live LSECs, where the step size is set to be smaller than 30 nm, n is the number of analysed fenestrations.

Supplementary Data 2 | ***Visualization of the actin fibres and fenestrations in live LSECs***

AFM visualization of the LSEC cytoskeleton structures, such as fenestrae associated cytoskeletal rings (FACR) and stress fibres is shown in **Supplementary Fig. 2 a, b, c**, where the images of the selected cell area are reconstructed from the same set of force-distance curves for 3 representative loading forces (for technical details see Methods): 100 pN, i.e., close to the contact point (**Supplementary Fig. 2a**); 200 pN, where both fenestrations and actin fibres can be identified (**Supplementary Fig. 2b**); and finally 800 pN, the maximum loading force used in the experiment (**Supplementary Fig. 2c**).

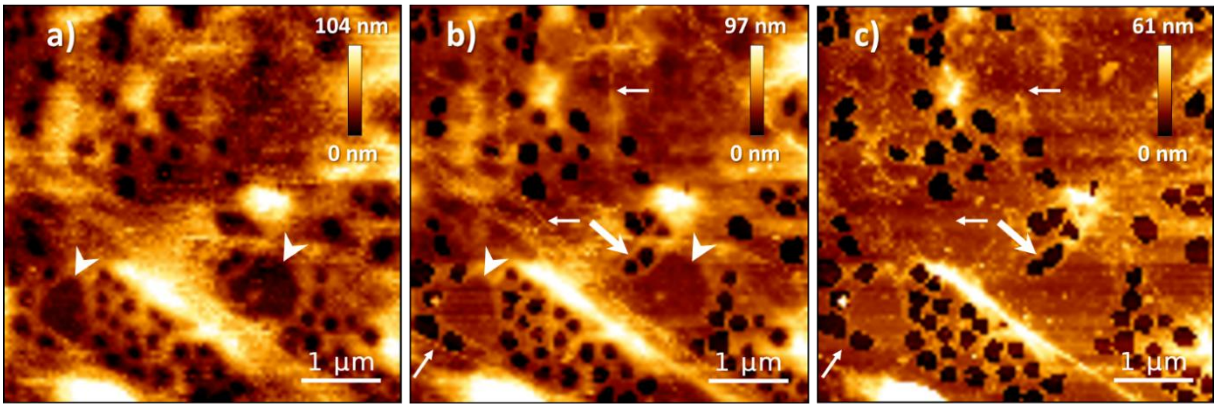

Supplementary Figure 2 | A selected 5.0 µm ×5.0 µm area of the live LSEC visualized with QI mode of AFM. Images were reconstructed for the following loading forces: **a)** 100 pN, **b)** 200 pN and **c)** 800 pN. Arrowheads indicate flat areas without fenestrations, which can be properly identified for higher loading forces (b and c). Small arrows indicate actin fibers which can be disclosed only in the narrow force window (b). Large arrows indicate an example of 2 fenestrations artificially fused due to application of the too high loading force. Image size and pixel resolution: 5.0 µm ×5.0 µm; 100×100.

It is seen that the ability of visualization of fenestrations and cytoskeleton structures, such as fenestrae-associated cytoskeletal rings (FACR) or stress fibers, is strongly related to the applied loading force and the speed of force-distance curve acquisition. The image reconstructed for the lowest loading force (close to the contact point) does not allow for proper identification of the fenestrations. Interestingly, flat cell membranes in the sieve plates (arrowheads in **Supplementary Fig. 2 a, b**) present similar contrast as the fenestrations.

However, use of higher loading forces in the range of 200 to 400 pNs results in proper visualization of both the fenestration structures and additionally the actin fibres (small arrows in **Supplementary Fig. 2**). At the other end, the loading force of 800 pN generates too strong interaction with the cell membrane leading to image artefacts appearing as merged fenestrations (see large arrow in **Supplementary Fig. 2 b, c**). Furthermore, flattening of the image and more uniform cell appearance occurs, affecting proper visualization of the cytoskeleton structures. The same effect is noticed if a too high speed (exceeding 80 µm/s) is applied. Using the speed of acquisition higher than 100 µm/s impedes proper control of the maximum loading force due to inertia of the cantilever.

Despite the use of high loading forces, fenestration structures remain unaffected by the tip interaction, which we prove by fixing the cells and scanning the same area again (**Supplementary Fig. 3**). Fixation stops the metabolism and increases stiffness of the cell by two orders of magnitude making AFM imaging much easier[1,2]. Observed fenestration diameters, however, are smaller after fixation due to shrinkage of the actin fibres in the fixation process, as described elsewhere[3,4]. Because of the large loading force some of the cell structures are squeezed or shifted under the tip pressure. Therefore, if the too high loading force is applied image artefacts are seen connected with an apparent merging of the fenestrations on the image. The appearance of the sieve plate before and after fixation is different because of the time elapsed between acquisition of the live cell image and the one after application of the fixation procedure.

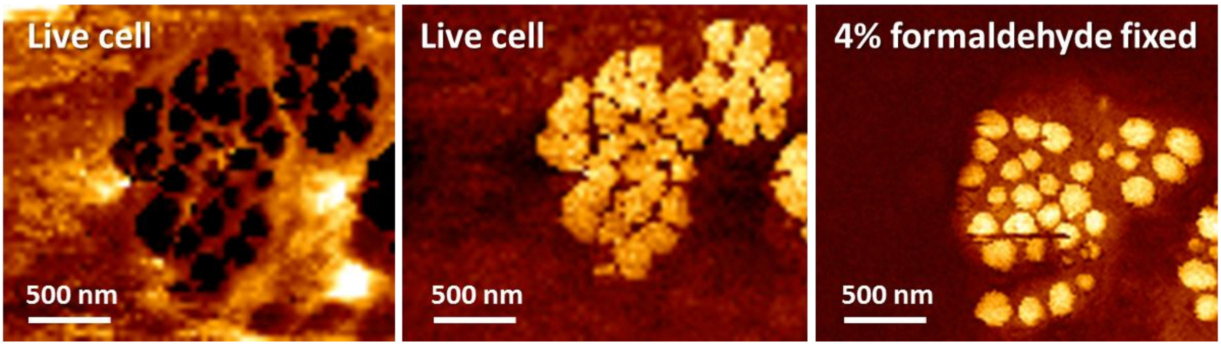

Supplementary Figure 3 | Selected area of the LSEC membrane visualized with a fast force-volume AFM imaging mode. Both, topography (left panel) and stiffness (central panel) images of the live LSEC present apparent merging of the fenestrations due to too high loading force (1.5 nN). After fixation with 4% formaldehyde the same area was scanned again immediately (right panel) using the same scanning conditions. It is clear that the FACR structure is preserved. Image size and pixel resolution: live cell - 2.5 μm ×2.3 μm, 65×60; fixed cell - 2.5 μm ×2.3 μm, 128×115.

Supplementary Data 3 | Dynamic changes in the porosity and number of fenestrations after treatment with cytochalasin B and antimycin A

Alterations in fenestration numbers and the cell porosity (percentage of the area covered with fenestrations to the total image area) are shown in **Supplementary Fig. 4**. We notice a rapid growth of the fenestration number after treatment with the cytochalasin B reaching plateau after about 10 minutes. In turn, Antimycin A treatment causes a steady decrease of the fenestration diameter and porosity leading to a complete loss of fenestrations after 60 minutes of the experiment.

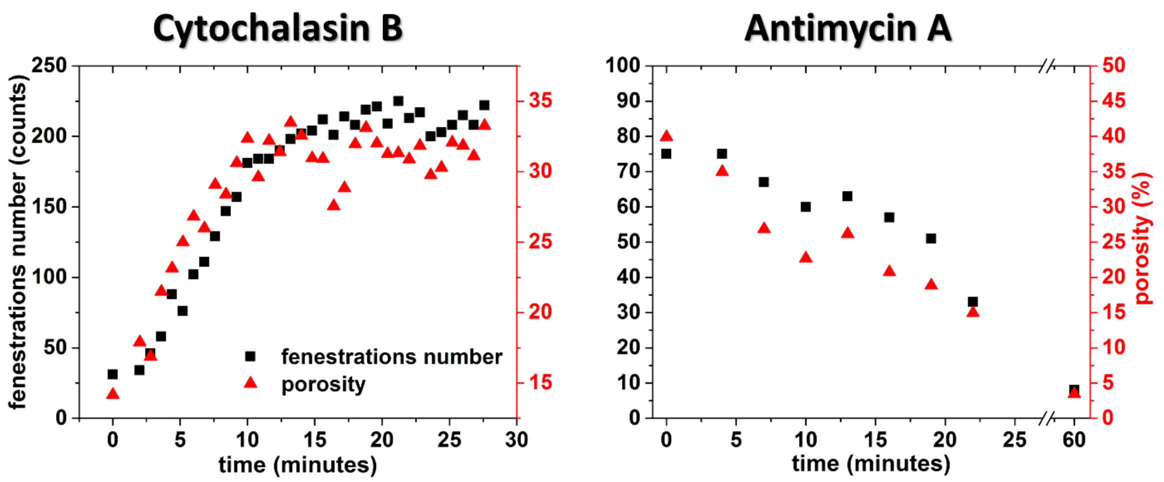

Supplementary Figure 4 | Time dependence of the fenestration number and porosity of LSEC membrane after treatment with 10 µg/ml cytochalasin B and 1 µg/ml antimycin A. Each point represents an independent QI AFM image shown in **Supplementary Animations 3 and 4**.

Supplementary Data 4 | ***Description of the supplementary animations***

Supplementary Animation 1

The rearrangement of the LSEC cytoskeleton presented in a 10 minutes long animation. Thin (<50 nm) and long (>1.0 µm) actin fibre structures are observed from one frame to another, rapidly changing their positions. Furthermore, formation and disappearing of the fenestrations are seen. Interestingly, two flat areas with diameters ~1.0 µm are observed without the fenestrated morphology. A single frame reconstructed for three selected loading forces is described in **Supplementary Fig. 2**.

The experiment was performed after 16 hours from the seeding of cells on the glass slide. The animation consists of 6 AFM topographic images. The imaged LSEC area is 5 µm ×

5 μm. Each image is reconstructed from 100×100 independent force-distance curves for the loading force of 200 pN. Acquisition of the single frame took 120 seconds.

Supplementary Animation 2

20 minutes long animation presenting dynamic changes of the selected LSEC area at the interconnection of two cells. The movement of the cell connected with rearrangement of the cytoskeleton is clearly shown. It is also seen that some fenestrations are appearing and disappearing when the thick stress fibre structures change their positions (upper left corner). Total number and diameter of fenestrations in the sieve plate, however, remain constant, allowing for determination of the fenestration diameter.

The experiment was performed after 19 hours from the seeding of cells on the glass slide. The animation consists of 14 AFM topographic images. The imaged LSEC area is 3.5 μm × 3.5 μm. Each image consist of 80×80 independent force-distance curves for the loading force of 400 pN. Acquisition of the single frame took 90 seconds.

Supplementary Animation 3

25 minutes long animation presenting selected LSEC area after the addition of Cytochalasin B. Rapid growth in the fenestration number is noticed after the addition of toxin. A newly formed sieve plate appears after 2 minutes. The fenestration forming centre can be distinguished after 6 minutes and moves in the direction of sieve plate expansion (for details see **Fig. 3** in the manuscript).

The experiment was performed after 20 hours from the seeding of cells on the glass slide. The animation consists of 34 AFM topographic images. The imaged LSEC area is 5.0 μm × 5.0 μm. Each image presenting the stiffness map is reconstructed from 100×100 independent force-distance curves. The maximum loading force is 1.0 nN. Acquisition of the single frame took 45 seconds.

Supplementary Animation 4

60 minutes long animation presenting selected LSEC area after the addition of antimycin A, an mETC complex III inhibitor. It is seen that the fenestration size is gradually decreasing leading to loss of fenestrations in time . Fenestrations with diameters smaller than 80 nm

just before the cell membrane fusion can be observed (for details see **Fig. 4** in the manuscript).

The experiment was performed after 16 hours from the seeding of cells on the glass slide. The animation consists of 9 AFM topographic images. The imaged LSEC area is 3.0 µm × 3.0 µm. Each image is reconstructed from 90×90 independent force-distance curves acquired for the loading force of 400 pN. Acquisition of the single frame took 3 minutes.

SI References

1. Zapotoczny, B. et al. Morphology and force probing of primary murine liver sinusoidal endothelial cells. J. Mol. Recognit. **30**, e2610 (2017). doi:10.1002/jmr.2610
2. Braet, F., Rotsch, C., Wisse, E. & Radmacher, M. Comparison of fixed and living liver endothelial cells by atomic force microscopy. Appl. Phys. A Mater. Sci. Process. **66**, 575–578 (1998). doi: 10.1007/s003390051204
3. Braet, F. et al. Comparative atomic force and scanning electron microscopy: An investigation on fenestrated endothelial cells in vitro. J. Microsc. **181**, 10–17 (1996). doi: 10.1046/j.1365-2818.1996.72348.x
4. Zapotoczny, B., Szafranska, K., Kus, E., Chlopicki, S. & Szymonski, M. Quantification of Fenestrations in Liver Sinusoidal Endothelial Cells by Atomic Force Microscopy. Micron **101**, 48-53 (2017). doi: 10.1016/j.micron.2017.06.005
