# Supplementary figures and images for: Atomic Force Microscopy Reveals the Dynamic Morphology of Fenestrations in Live Liver Sinusoidal Endothelial Cells
Source: Sci Rep. 2017 Aug 11;7:7994. doi: 10.1038/s41598-017-08555-0 (PMC5554186; doi:10.1038/s41598-017-08555-0)

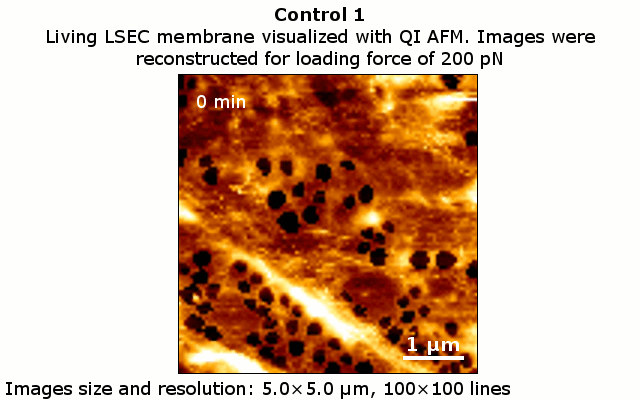

Supplement: Supplementary file 2 — Supplementary Animation 1 [file 41598_2017_8555_MOESM2_ESM.gif]

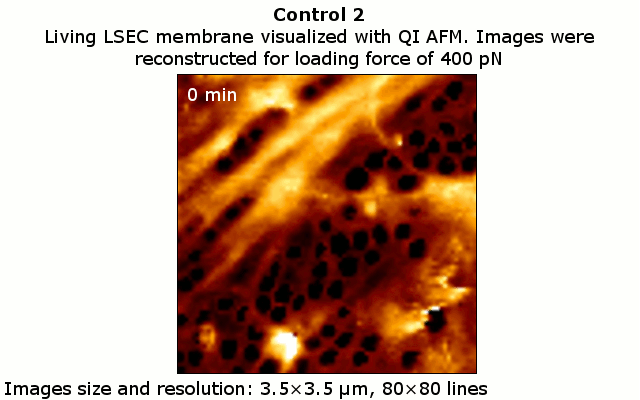

Supplement: Supplementary file 3 — Supplementary Animation 2 [file 41598_2017_8555_MOESM3_ESM.gif]

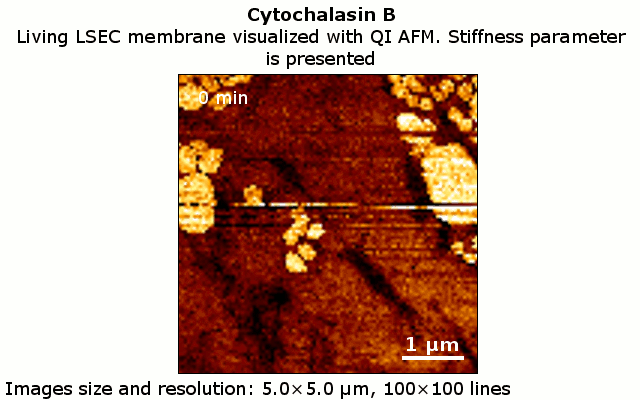

Supplement: Supplementary file 4 — Supplementary Animation 3 [file 41598_2017_8555_MOESM4_ESM.gif]

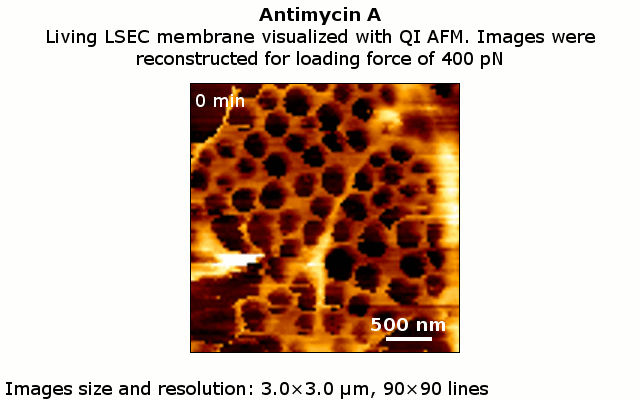

Supplement: Supplementary file 5 — Supplementary Animation 4 [file 41598_2017_8555_MOESM5_ESM.gif]
